# Probing the Release of Bupropion and Naltrexone Hydrochloride Salts from Biopolymeric Matrices of Diverse Chemical Structures

**DOI:** 10.3390/polym13091456

**Published:** 2021-04-30

**Authors:** Angeliki Siamidi, Aikaterini Dedeloudi, Marilena Vlachou

**Affiliations:** Division of Pharmaceutical Technology, Department of Pharmacy, School of Health Sciences, National and Kapodistrian University of Athens, 15784 Athens, Greece; asiamidi@pharm.uoa.gr (A.S.); dedeloud@pharm.uoa.gr (A.D.)

**Keywords:** bupropion, naltrexone, modified release, ulvan, matrix tablet, biopolymers

## Abstract

In the last decades, the notion of including excipients in the formulations, as inert substances aiding production processes, has changed and they are recently viewed as multifunctional discrete entities. It is now well documented that excipients serve several roles, spreading from the stabilization and modified release, to providing biocompatible properties and targeting moieties. The aim of this study was to develop matrix-based oral drug delivery systems of bupropion hydrochloride (BUP·HCl) and naltrexone hydrochloride (NTX·HCl), suitable for releasing these active substances in a modified manner, providing a stable level of drug release, which is simultaneously therapeutically effective and non-toxic, thus reducing side effects, after a single dose administration, throughout the gastrointestinal tract. The new formulations, employing hydroxypropylmethycellulose (HPMC K15M) (a cellulosic polymer, which, generally hydrates to form a gelatinous layer that is critical to prevent wetting and rapid drug release from the matrices), poly(methacylic acid-*co*-ethyl acrylate) 1:1 (Eudragit^®^ L100-55: effective for site specific drug delivery in intestine), poly(ethylene oxide) (PEO) (7 × 10^6^: a high molecular weight polymer, water-soluble, in micro-granular powder form), as the rate controlling polymers, were chosen to lead to a “soothing out” release pattern of these drugs, at 0 ≤ t ≤ 120 min. Moreover, the release of the two drugs from the ulvan-based tablets, was found to follow the desired profile, throughout the entire course of the dissolution experiments.

## 1. Introduction

**Scheme 1:**
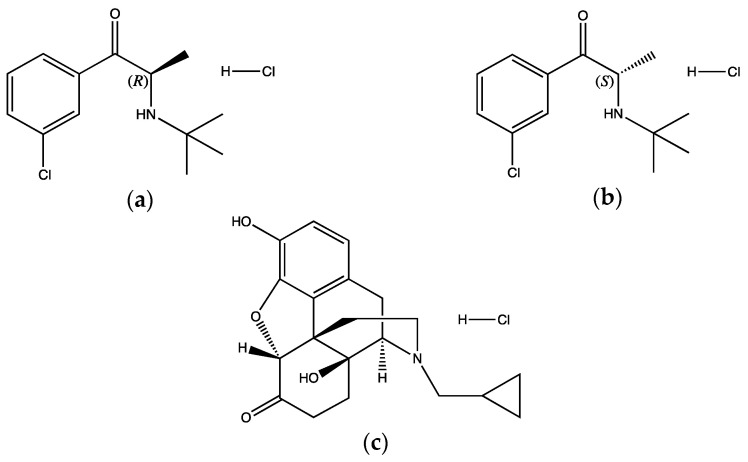
Chemical structures of: (**a**) (2*R*)-bupropion.HCl, (**b**) (2*S*)-bupropion HCl and (**c**) naltrexone HCl.

Bupropion hydrochloride, (±)-1-(3-chlorophenyl)-2-[(1,1-dimethylethyl)amino]-1-propanone, hydrochloride) (BUP·HCl), is a BCS Class I drug (high solubility and high permeability) [1,2] with a low molecular weight (molecular weight of its HCl salt = 276.2 g/mol), which has been in therapeutic use, as an antidepressant, since 1989 [3]. As far as the physicochemical properties of BUP·HCl are concerned, it is characterized by two crystalline polymorphic structures, which appear to be enantiotropically related [4] and has an asymmetric center, resulting to two enantiomers, the (2*R*)-bupropion and the (2*S*)-bupropion (Scheme 1). Although the pure enantiomers of BUP·HCl have been successfully synthesized, rapid racemization occurs in aqueous solution. Therefore, this drug is available in the market, as a racemic mixture [5,6].

Pharmacologically, BUP·HCl, which is an aminopropiophenone, is characterized as a dopamine/*nor*epinephrine reuptake inhibitor (NDRI) [7]. It is a selective inhibitor of neuronal reuptake of catecholamines (*nor*adrenaline and dopamine), with negligible action of indolamine reuptake (serotonin). It is also a potent, non-competitive antagonist of central nicotinic receptors. Nevertheless, the exact mechanism of action of BUP·HCl remains unclear [5]. It was originally classified as an “atypical” antidepressant, due to its different effects, from conventional antidepressants, such as the monoamine oxidase inhibitors, the tricyclic antidepressants, and the selective serotonin reuptake inhibitors [8]. BUP·HCl is most commonly used to confront major depression, seasonal affective disorder (SAD), smoking cessation, attention deficit hyperactivity disorder and eating disorders [9]. Moreover, it is extensively metabolized in humans, by liver enzymes (mainly CYP2B6), into three metabolites: hydroxybupropion, *threo*-hydroxybupropion and *erythro*-hydroxybupropion, which have been suggested to contribute and/or be responsible for its antidepressant properties. Hydroxybupropion and *threo*-hydroxybupropion are the major metabolites in humans, while the *erythro*-hydroxybupropion, is formed in smaller amounts [10].

Immediate-release BUP·HCl (Wellbutrin^®^ 75 mg, 100 mg) is commercially available since 1989 and is usually a well-tolerated antidepressant. However, seizures appeared in approximately 0.4% of patients treated at daily doses up to 450 mg. In order to reduce the adverse side effects, sustained-release Wellbutrin^®^ SR (100 mg, 150 mg, 200 mg and 300 mg) has been developed. Wellbutrin^®^ SR tablets comprise of an hydroxypropylmethycellulose HPMC-based matrix core containing, cysteine hydrochloride, as a stabilizer, and film coating [11,12,13].

Naltrexone [(4*R*,4*aS*,7*aR*,12*bS*)-3-(cyclopropylmethyl)-4*a*,9-dihydroxy-2,4,5,6,7*a*,13-hexahydro- 1*H*-4,12-methanobenzofuro[3,2-*e*]isoquinolin-7-one] (NTX·HCl, Scheme 1) is an opioid receptor antagonist and a synthetic derivative of oxymorphone [14,15]. It has the potential to bind with *κ*, *δ* and *μ* receptors in the central nervous system (CNS). However, NTX·HCl shows a higher affinity in binding with *μ* receptors and acts competitively with the endogenous opioids of the CNS [16]. It is used in pharmacotherapy against a plethora of human disorders, with the most significant being, alcohol and heroin dependence, schizophrenia, obesity and eating disorders [17,18]. Naltrexone is classified as a BCS IV drug (low solubility and low permeability) [19] and is absorbed rapidly after following *per os* administration. The metabolism of NTX·HCl occurs in the liver, producing an active metabolite, the 6-*β*-naltrexol; the elimination of naltrexone and 6-*β*-naltrexol is taking place primarily in the kidneys [20,21,22].

Biopolymers, when used as pharmaceutical excipients, play an important role in drug delivery technology. Polymeric drug delivery systems enable the introduction of a therapeutic substance into the body, not only by improving its safety and efficacy, but also by controlling the rate, time, and site of release of the drug in the body. Polymers can provide modified release of both hydrophilic and hydrophobic drugs. The mechanisms of drug release can be linked to drug diffusion, dissolution and degradation of the matrix system, where the drug is embedded.

The development of drug delivery systems, that are based on natural and/or synthetic polymers, have rendered high expectations in the pharmaceutical field. Natural polymers can be of various sources, marine polysaccharides, being amongst them. Such a biopolymer, of marine origin, is ulvan, which is isolated from green algae (*Ulva lactuca and Enteromorpha*), and is comprised of a group of highly sulfated polysaccharides with acidic properties, mainly composed with rhamnose 3-sulfate, xylose 2-sulfate, glucuronic acid and iduronic acid [23]. Its unique chemical structure is responsible for several biological and therapeutic activities, as, for example, antiviral, antibacterial, immunostimulatory, antioxidant, antihyperlipidemic and anticoagulant [24].

Moreover, it is bio-compatible, non-toxic, bio-degradable and stimuli-responsive polymer, characterized as a suitable and optional excipient in designing and formulating drug delivery systems with a release that can be effectively controlled [25]. Ulvan is a multifunctional and multi-responsive polysaccharide, thus the structural organization of its polymeric chains and consequently its gel formation can be modified in pH-, ion- and temperature-modified conditions, defining the final release rate of the active pharmaceutical ingredient (API) [26,27]. In particular, when ulvan is present in a low or a neutral pH aqueous medium is immediately transformed to a condensed bead-like conformation due to the hydrophobic properties of rhamnose [28]. Moreover, in a simultaneous presence of counterions, ulvan polymeric chains immediately form aggregates, characterized by low viscosity and rheological properties, influencing considerably the release rate of drug delivery systems [29]. Consequently, in drug delivery, ulvan can alter the release of the therapeutic agent by forming either physical and/or chemical hydrogels with multifunctional properties [30].

The aim of the present study is to communicate our findings on the BUP·HCl and NTX·HCl modified release from solid dosage forms, using polymers of diverse origins, *en route* to our future task to develop oral compounded formulations of these two drugs, capable of confronting the obesity menace.

Modified release formulations are advantageous, as they maintain the concentration of the administered drugs within the therapeutically effective range, thus decreasing the dosing intervals [31,32]. BUP·HCl is, as already mentioned, an antidepressant used for smoking cessation and to treat a variety of conditions, including depression and other mental/mood disorders. Antidepressants can help in preventing suicidal attempts and provide other important benefits [33]. On the other hand, NTX·HCl is a drug substance mainly used in alcohol/heroin dependence and for the treatment of problems, such as eating behavior disorders (e.g., bulimia and anorexia nervosa), obesity and erectile dysfunction [15,17]. Both of these active substances could benefit from modified release tablet formulations, as the commercially available BUP·HCl product, Wellbutrin^®^ XR, exhibits a satisfactory release (almost 67%) in the intestinal environment, but, no release at the stomach pH. The same holds true for naltrexone hydrochloride, which to the best of our knowledge, is mainly administered, as a formulation for depot injection, containing 380 mg of the medication per vial (Vivitrol^®^) [34]. The new formulations, employing hydroxypropylmethycellulose (HPMC K15M) (a cellulosic polymer, which, generally hydrates to form a gelatinous layer that is critical to prevent wetting and rapid drug release from the matrices), poly(methacylic acid-*co*-ethyl acrylate) 1:1 (Eudragit^®^ L100-55: effective for site specific drug delivery in intestine), poly(ethylene oxide) (PEO) (7 × 10^6^: a high molecular weight polymer, water-soluble, in micro-granular powder form) and ulvan, as the rate controlling polymers, could lead to a “soothing out” release pattern of these drugs, at 0 ≤ t ≤ 120 min.

## 2. Materials and Methods

### 2.1. Materials

NTX·HCl (MW: 377.87 g/mol, >98.0% purity) and BUP·HCl (MW: 276.20, >98.0% purity) were purchased from Tokyo Chemical Industry (Japan). Wellbutrin^®^ XR 150 mg (Lot.No. 19F007, GlaxoSmithKline, Industrial & Commercial S.A.) and Wellbutrin^®^ XR 300 mg (Lot.No. F78H, GlaxoSmithKline, Industrial & Commercial S.A.), were donated from a community pharmacy store (T. Papasandas, Athens, Greece). HPMC K15M and PEO (MW: 7 × 10^6^ g/mol) were supplied from Sigma-Aldrich (Steinheim, Germany). Eudragit^®^ L100-55 was obtained from Rohm GmbH Pharma Polymers (Darmstadt, Germany). Ulvan (Cat. No. YU11689) was purchased from Carbosynth^®^ Ltd. (Berkshire, UK) and magnesium stearate was supplied by Riedel-De Haen (Hannover, Germany). All chemicals were of reagent grade and used in this study without further purification.

### 2.2. Methods

#### 2.2.1. Preparation of BUP·HCl and NTX·HCl Modified Release Tablets

**Figure 1:**
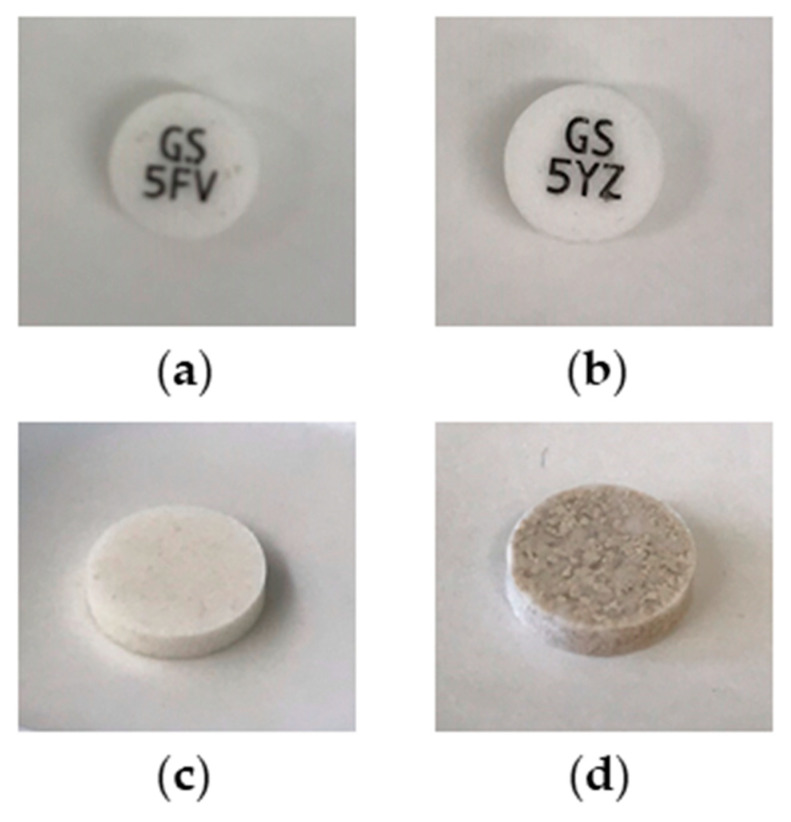
Tablet formulations of: (**a**) Wellbutrin^®^ XR 150 mg, (**b**) Wellbutrin^®^ XR 300 mg, (**c**) bupropion·HCl and (**d**) naltrexone·HCl.

Matrix tablets of the APIs (BUP·HCl and NTX·HCl) were prepared by blending and compressing them with a variety of excipients (*vide* Table 1, Figure 1). In detail, the APIs and excipients (HPMC, PEO, Eudragit^®^ L100-55 and ulvan) were blended in a laboratory scale powder blender at 32 rpm for 10 min. Afterwards, the lubricant, magnesium stearate, was added and mixing was continued for 5 more minutes. The powder mixture was accurately weighed (200 mg), loaded on a 10 mm diameter dye and directly compressed, using a hydraulic press (Maassen type, MP 150); loading compression force (5.00–5.50 tons).

#### 2.2.2. Post Compression Parameters

*Thickness Test***:** The thickness of the tablets was measured using a Vernier calipers scale [35].

*Hardness Test***:** The hardness of the tablets was determined using an Erweka hardness tester (Erweka, typeTBH28). The force applied was equal to breaking the tablet in a diametric compression. The surface hardness of each tablet is expressed in N [35].

#### 2.2.3. In Vitro Dissolution Studies

The dissolution tests were carried out in a tablet dissolution test apparatus USP type II (Pharmatest, Hainerp, Germany) (paddle method). The experiments were performed in two different aqueous media: for the first 2 h, 450 mL of 0.2 M HCl solution (pH 1.2) was used to simulate the stomach pH, and to that 450 mL of 0.14 M K_2_HPO_4_ solution (pH 9) was then added, to have the required composition of the next phase, which simulates the enteric pH (pH 6.8, final volume 900 mL). [36].

The temperature of the dissolution medium was maintained at 37 ± 0.5 °C. The tablet was placed in the bottom of a vessel, equipped with paddles, under sink conditions, and the apparatus was operated at 50 rpm. Samples were withdrawn in predetermined time intervals and the withdrawn volume of the medium was replenished. The withdrawn samples were filtered and analyzed in a Perkin–Elmer UV spectrophotometer (Norwalk, CT) at λ_max_= 251 nm (for BUP·HCl) and at λ_max_= 281 nm (for NTX·HCl). The % dissolution curve was determined according to the calibration curve of the corresponding API. All experiments were carried out six-fold (*n* = 6).

#### 2.2.4. Methods to Compare Dissolution Profiles

**Figure 2:**
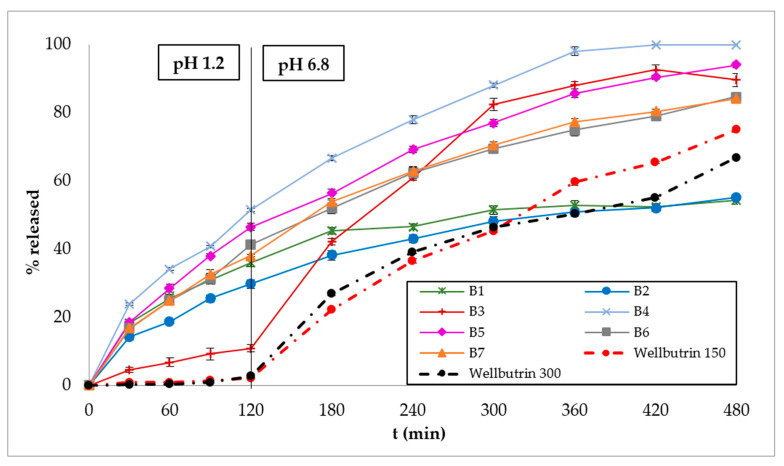
In vitro % release of bupropion hydrochloride (BUP·HCl) and the commercially available product (Wellbutrin^®^ XR 150 and 300 mg) vs. time Figure 1. 2 (0–120 min) and at pH 6.8 (120–480 min). The results denote the mean value (*n* = 6, SD < 2).

**Figure 3:**
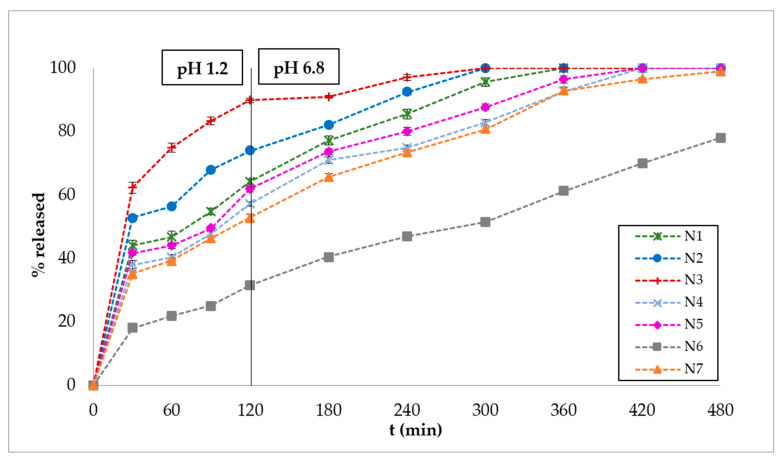
In vitro % release of naltrexone hydrochloride (NTX·HCl) vs. time from different tablet formulations at pH 1.2 (0–120 min) and at pH 6.8 (120–480 min). The results denote the mean value (*n =* 6, SD < 2).

In order to compare the dissolution profiles, graphs of % drug release vs. time were constructed (Figure 2 and Figure 3) and the Dissolution Efficiency % [D.E. (%)] value was estimated. According to Khan (1975) [37], D.E. (%) is a useful parameter for the evaluation of dissolution in vitro and is calculated from the following equation:

(1) $$D.E.\;\left(\%\right)=\frac{\int_{t_{1}}^{t_{2}}y\;dt}{y_{100}\left(t_{2}-t_{1}\right)}\times 100$$

where, y is the percentage of dissolved product and D.E. (%) the area under the dissolution curve between time points t_1_ and t_2_, expressed as a percentage of the curve at maximum dissolution y_100_, over the same time period. Dissolution efficiency, which takes into account the dissolution profile as a whole, was employed to interrelate dissolution with the other variables used in this study.

Furthermore, t_20%_, t_50%_ and t_90%_, as well as the mean dissolution time (MDT) values were estimated. The t_20%_, t_50%_ and t_90%_, values refer to the time, where the 20%, 50% and 90% of the active substance is released. MDT is the value used to characterize the drug release rate from a dosage form and the following is used to derive an estimate of MDT from experimental dissolution data [38]:

(2) $$MDT=\frac{ABC}{W_{\infty }}$$

where, W_∞_ is the maximum amount of the drug substance that is dissolved, and ABC is the area between the drug dissolution curve and its asymptote.

The in vitro release data were fitted to the Korsmeyer–Peppas equation:

(3) $$\frac{M_{t}}{M_{\infty }}=Kt^{n}$$

where, *M_t_* and *M_∞_* denote the absolute cumulative amount of drug released at time *t* and infinite time, respectively, *k* the release rate constant and *n* the diffusion coefficient. This equation is only valid for the first 60% of the fractional release [39]. In the case of cylindrical tablets, *n* ≤ 0.45 corresponds to a Fickian diffusion release (case I diffusional), 0.45 < *n* < 0.89 to an anomalous transport, and *n* = 0.89 to zero order (case II) release kinetics.

#### 2.2.5. Anova-Based Analysis

D.E. (%) results were expressed as the mean ± standard deviation (SD) and analyzed using the Tukey’s post hoc test for this ANOVA test (*p* < 0.05 for significantly different results).

#### 2.2.6. Model-Independent Analysis of Dissolution Profiles

Comparison indices, such as the difference factor (f_1_) and similarity factor (f_2_), were used to compare the dissolution profiles of the APIs [40,41].

#### 2.2.7. Polymer Erosion Studies

Polymer erosion studies were also carried out, as the degree of polymer erosion is a characteristic, which may affect, considerably, the polymer behavior and therefore, drug release. Pre-weighed matrix tablets were introduced into the dissolution apparatus, under the standard set of conditions, specified for the release rate studies. The tablets were withdrawn using a small basket at selected time intervals and placed in an oven at 45 °C, for 24 h. The dried tablets were cooled down in a desiccator and then weighed. This process was repeated until a constant weight was achieved (final dry weight). Six different tablets were measured, at each time point, and fresh tablets were used for each individual time point. The extent of erosion (E) was determined by the following equation:

(4) $$Ε\%=\frac{100\left(W_{i}-W_{f}\right)}{W_{i}}$$

where, *W_i_* is the initial starting dry weight and *W_f_* is the final dry weight of the same dried and partially eroded tablet, respectively [42].

#### 2.2.8. Disintegration Studies

The disintegration process is specifically critical for immediate-release dosage forms, which are designed to fully disintegrate and dissolve upon exposure to physiological fluids within a short period of time (2.5 to 10 min). In the present study, the tablets were designed to provide a modified release profile, and therefore, the time to disintegrate would be much higher than 10 min, according to excipient properties, which allow tablet disintegration in a neutral pH aqueous media [43,44].

## 3. Results

Tablet formulations of Wellbutrin^®^ XR 150 mg, Wellbutrin^®^ XR 300 mg, bupropion HCl and naltrexone HCl are depicted in Figure 1.

### 3.1. Post Compression Parameters

*Thickness Test*: In all cases, tablets of 10 mm diameter and thickness (2 ± 0.01 mm) were produced.

*Hardness Test*: The surface hardness of each tablet is expressed in N, in the range of 96.30–123.37 N.

The physical properties of the new formulations were considered, in all cases, acceptable.

### 3.2. In Vitro Dissolution Studies

The dissolution profiles of both BUP·HCl and NTX·HCl are depicted in Figure 2 and Figure 3, respectively.

The kinetic release properties of the developed formulations (B1-B7, N1-N7), Wellbutrin^®^ XR 150 and 300 mg, are reported in Table 2. The terms t_20%_, t_50%_ and t_90%_ refer to the time when 20%, 50% and 90%, respectively, of the dissolution that has been achieved. D.E.% refers to % dissolution efficiency and *n* denotes the release kinetics according to the power law.

It is apparent from the data presented in Figure 2, that the % release of BUP·HCl from formulations B1-B7, with the exception of B3 (marginally higher at pH 1.2), was higher than that of the market drug, Wellbutrin XR^®^ 150 and 300, at both pHs (1.2 and 6.8).

**Scheme 2:**
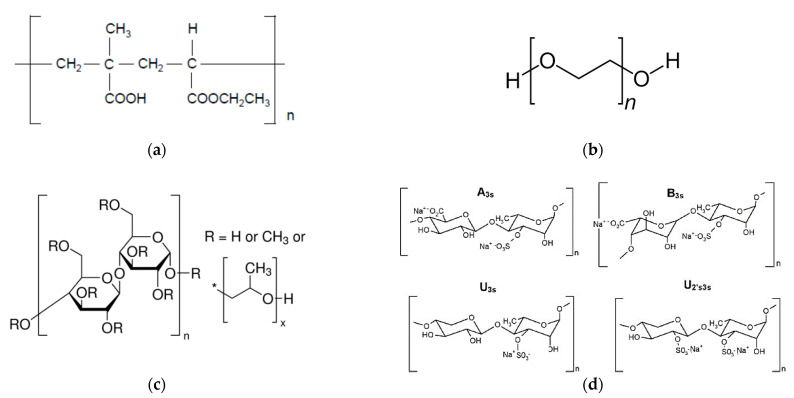
Chemical structure of: (**a**) Eudragit^®^ L100-55, (**b**) poly(ethylene oxide) (PEO), (**c**) hydroxypropylmethycellulose (HPMC K15M) and (**d**) structure of the main repeating disaccharides in *Ulva* ulvan: ulvanobiuronic acids A_3S_ and B_3S_ and ulvanobioses U_3S_ and U_2′S,3S_ [29].

Specifically, the % release of BUP·HCl from formulation B6, which contained PEO, was higher (41.29%; pH 1.2) than that from formulation B3 (10.87%; pH 1.2), which contained Eudragit^®^ L100-55. An enhanced release of BUP·HCl from formulation B3 was noticed only at pH 6.8. Albeit the fact that, at this pH, BUP·HCl was not protonated and thus its aqueous solubility was reduced, the presence of Eudragit^®^ L100-55 (Scheme 2) permitted the facile water penetration into these tablets, at pH 6.8, leading to an increased release of this API (B3: 89.63%; B6: 84.73% at 480 min) [42]. The % release of BUP·HCl from formulations B4 and B5 was higher (71.03% and 46.50%, respectively) than that from formulation B6 (41.29%), at pH 1.2. At pH 6.8, the release of BUP·HCl from the tablets of formulation B5 resembles the release from formulation B3, at 300 min and thereafter.

The substitution of Eudragit^®^ L100-55 (formulation B3) in the formulations B4 and B5, with PEO, especially from B4, which contained a higher amount of PEO (37.5%), in comparison to B5 (24%), led to an increase of the total % drug release (100% and 94.02%, compared to 89.63%). PEO (Scheme 2) is the most widely used excipient in controlled release matrix tablets, owing to its aqueous solubility, availability in a range of molecular weight/viscosity grades and unique swelling/erosion properties, which can be utilized in modulating drug release profiles [11,42].

The replacement of Eudragit^®^ L100-55 (formulation B3) in formulations B1 and B2, with HPMC, especially in B1 that contained a higher amount of HPMC (37.5%) in comparison to B2 (24%), led to a noteworthy release of BUP·HCl, at pH 1.2 (B1: 35.98%; B2: 29.72%, at 120 min), and to a sustained release, thereafter (B1: 54.29%; B2: 55.13%, at 480 min). It seems these HPMC K15M-based formulations were able to control the release of the drug to a higher extent than those containing Eudragit^®^ and PEO based formulants. This could be attributed to the high viscosity and density of HPMC K15M (Scheme 2), which forms a rigid gel upon contact with the aqueous media, thus controlling the delivery of the highly water-soluble drug (BUP·HCl) [45].

The drug release from the formulations B1 and B2 (that contained 37.5% HPMC and 24% HPMC, respectively) showed a decrease in relation to the drug release from formulations B4 and B5 (that contained 37.5% and 24% PEO, accordingly) (D.E.% B1: 39.56, B2: 36.53 and B4: 71.03, B5: 62.81). In previous papers, the same trend was observed when HPMC K15M and PEO including tablets of BUP·HCl, were compared [46]. It has been adequately documented that in swellable matrices, the release of the drug is sustained by one or more of processes, including the diffusion of water into the matrix, the swelling, due to the hydration of matrix or the relaxation of polymer chains (often referred as Case II transport), a diffusion process of the drug, through the existing pores or erosion of the matrices [47,48,49]. The release rate of BUP·HCl was faster through the PEO containing matrices than that of HPMC K15M; this may be due to the overall erosion rate of PEO, which is faster than that of HPMC K15M.

The drug release from the formulations B5, B4 and B6, which contained 24%, 37.5% and 61.5% PEO, respectively (D.E.% B5: 62.81, B4: 71.03 and B6: 55.76) showed a decrease when the % PEO was increased. In previous reports, the same trend was observed; the decrease in drug release with increasing PEO content may be attributed to the increased viscosity of the gel layer, as well as, the formation of a gel layer with a thicker diffusional layer [50,51,52,53].

The tablets containing ulvan (24%) (formulation B7), showed a relatively higher drug release (B7: 38.08%, at 120 min and 84.21% at 480 min) than that of the market drugs (Wellbutrin^®^ XR 150: 2.35% at 120 min and 75.09% at 480 min; Wellbutrin^®^ XR 300: 2.62% at 120 min and 66.85% at 480 min) which contained polyvinylalcohol and glyceryl dibehenate inside the tablets and ethylcellulose, polyethylenoglycol 1450, Povidon Κ90, methacrylic acid, ethyl acrylate copolymer dispersion (Eudragit^®^ L30 D-55), silicon dioxide and triethyl citrate, as a coating. Ulvan, conversely to the commonly used algae, sodium alginate, has an unusual chemical composition, and a regular structure combining. The marine product, used in the present study, was highly sulphated and contained rhamnose 3-sulphate, xylose, xylose 2-sulphate, glucuronic acid and iduronic acid residues. Thus, in the acidic environment, a number of ulvan’s functionalities remained charged, facilitating that way the dissolution of BUP·HCl (approximately 38%). It is interesting to note that at pH 6.8, this dissolution enhancing effect of ulvan was maintained. BUP·HCl reached an 84.21% release from tablets containing ulvan (B7) at 8 h. At this pH, all the sulfate and carboxylate groups of ulvan were ionized, thus facilitating the BUP’s release. Apart from the pH value and the functionalities of ulvan, which affected the dissolution of bupropion, the amorphous shape and size of ulvans [54], also contributed to BUP’s enhanced release. Moreover, ulvan’s particles have a sponge-like shape with large cavities through which the molecules of the aqueous medium penetrate faster and deeper [Scheme 2].

The kinetics release data, obtained via the Korsmeyer–Peppas equation (Table 2), revealed that the release of BUP·HCl, from the formulations tested, followed in most cases (B4 (*n =* 0.61), B5 (*n =* 0.61), B6 (*n =* 0.66), B7 (*n =* 0.67) Wellbutrin^®^ XR 150 mg (*n =* 0.51) and Wellbutrin^®^ XR 300 mg (*n =* 0.75)), a non-Fickian pattern (0.45 < *n* < 0.89, anomalous diffusion), i.e., a combined mechanism of pure diffusion and Case II transport [55].

Factors f1 and f2 were calculated, by using the respective equations, and showed that apart from formulation B3, which contained Eudragit^®^ L100-55, all the other formulations differed from those calculated for Wellbutrin^®^ XR (150 mg and 300 mg) (Table S1, Supplementary Materials); this was in agreement with the release data presented above. These results were also corroborated by the ANOVA tests (Tables S2 and S3 in Supplementary Materials) on the respective D.Es.%. 

The dissolution curves of NTX·HCl (N1-N7) from the matrix tablets at both pHs (1.2 and 6.8) are depicted in Figure 3. In detail, the % release of NTX·HCl from formulation N6, which contained PEO (95%), was lower (77.90%) than that from formulation N3 (100%), involving Eudragit^®^ L100-55 (95%), at both pHs, 1.2 and 6.8. This finding, which was the opposite of the that noticed in the respective formulation of BUP·HCl, could be attributed to the fact that BUP·HCl, *albeit* being a much more water-soluble compound (solubility: 312 mg/mL) [5,56] than NTX·HCl (solubility: 100 mg/mL) [57], was used in a much higher quantity (37.5%), in all formulations tested, than NTX·HCl (4%). The same trend was noticed and at pH 6.8. This was expected, as the much higher quantity of Eudragit^®^ L100-55 in all NTX·HCl formulations, lead to an enhancement of its release, because in the formulation N3, which involved the pH dependent, water soluble polymer, Eudragit^®^ L100-55, a facile water penetration into the tablet was permitted and consequently the hydration of the formulation system, leading to a sustained drug dissolution profile [58]. 

However, the % release of NTX from formulations N4 (Eudragit^®^ L100-55 37%; PEO 58%) and N5 (Eudragit^®^ L100-55 58%; PEO 37%) was higher (100% release in both cases) than that from formulation N6 (PEO 95%) (77.90%), at both pH’s. The release from the formulation N4 resembled the release from formulation N5 (D.E.% N4: 72.21 and N5: 75.28). The substitution of Eudragit^®^ L100-55 (formulation N3) in formulations N4 and N5, with PEO, especially N4 that contained a higher amount of PEO (58%) in comparison with N5 (37%), lowered the total % drug release. As previously mentioned, PΕOs are swellable polymers, capable of forming strong hydrogen bonds with the water molecules, resulting to high water uptake and modified release [42]. 

The replacement of Eudragit^®^ L100-55 (formulation N3) in formulations N1 and N2 with HPMC K15M, especially N1, which contained a higher amount of HPMC K15M (58%) in comparison with N2 (37%), lowered the total % drug release (D.E.% N1: 78.74 and N2: 83.67). It seemed these HPMC K15M-based formulations were able to control the release of the drug to a higher extent than those containing the Eudragit^®^ and PEO formulants. This could be attributed to the high viscosity and density of HPMC K15M, leading to a rigid gel formation, upon contact with the aqueous media, thus controlling the delivery of the sparingly water-soluble drug (NTX·HCl) [45].

The drug release from the formulations N1 and N2 (containing 58% HPMC and 37% HPMC, respectively) showed a small increase in relation to the drug release from formulations N4 and N5 (58% and 37% PEO, respectively) (D.E.% N1: 78.74, N2: 83.67 and N4: 72.21, N5: 75.28). This was in agreement with the findings, reported in US 8,916,195B2 patent, on HPMC and PEO involving NTX tablets [59].

The tablets containing ulvan (37%) (formulation N7), showed a relatively higher drug release (N7: 52.80%, at 120 min and 98.97% at 480 min; D.E.%: 70.23), when compared with N6 (N6: 31.39%, at 120 min and 77.90% at 480 min; and D.E.%: 45.60). At pH 1.2, the sulfate groups of ulvan remain uncharged, conversely to its carboxylate groups. This partial ionization of ulvan leaded to an enhancement of NTX release from the N7 tablets, compared to the non-ulvan containing tablets N6.

It was interesting to note that at pH 6.8, this dissolution enhancing effect of ulvan was maintained. At this pH, apart from the sulfate groups of ulvan, its carboxylates are also present in the ionized form, and this led to an enhanced NTX release from the N7 formulations. The substitution of Eudragit^®^ (formulation N4) in formulation N7 (D.E.% N4: 72.21 and N7: 70.23) with ulvan slightly lowered the % drug release.

The kinetics release data, obtained via the Korsmeyer–Peppas equation (Table II), revealed that the release of NTX from the formulations studied, followed in the cases of N6 (*n*: 0.56) a non-Fickian pattern (0.45 < *n* < 0.89, anomalous diffusion), i.e., a combined mechanism of pure diffusion and Case II transport [55], whilst in the case of N7 (*n*: 0.39), which included the ulvan polymer, it approached the Fickian pattern.

### 3.3. Polymer Erosion Studies

**Figure 4:**
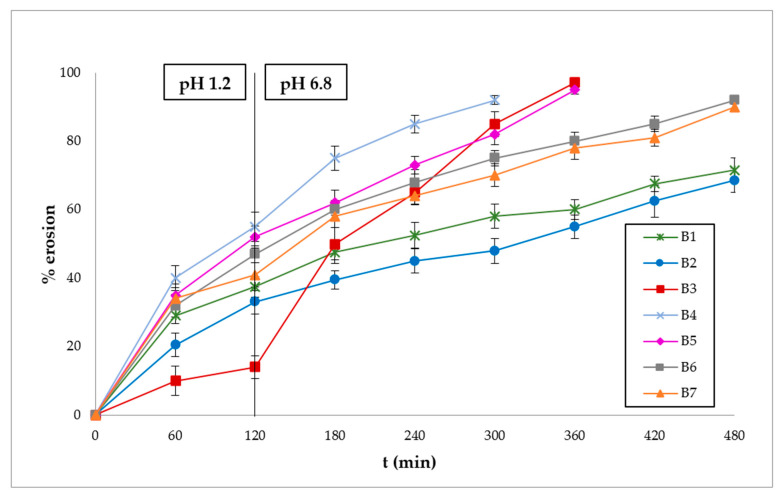
% Erosion of BUP·HCl tablets formulations vs. time at pH 1.2 (0–120 min) and at pH 6.8 (120–480 min). The results denote the mean value (*n =* 6).

**Figure 5:**
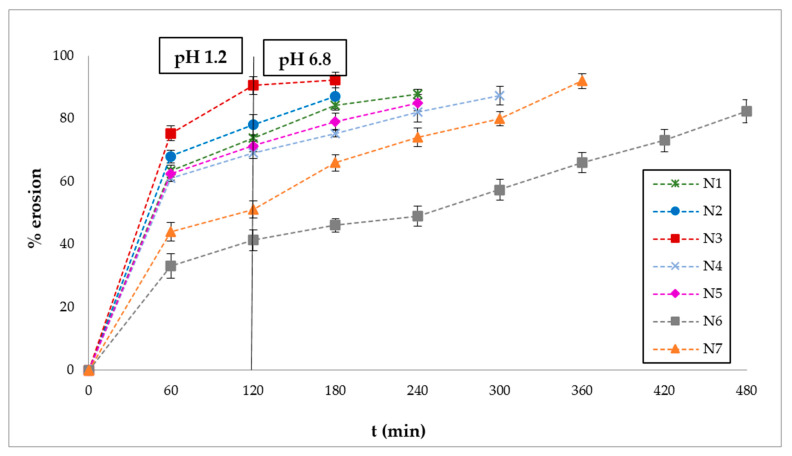
% Erosion of NTX·HCl tablets formulations vs. time at pH 1.2 (0–120 min) and at pH 6.8 (120–480 min). The results denote the mean value (*n =* 6).

The erosion profiles of both BUP·HCl and NTX·HCl are depicted in Figure 4 and Figure 5, respectively.

Optical observation of B1 and B2 tablet formulations (containing 37.5% and 24% HPMC, respectively) during the dissolution and erosion experiments showed an increase of the gel network that delayed the water penetration into the glassy core [60]. Therefore, it could be assumed that the mechanism of these matrix systems was controlled by diffusion rather than erosion [61]; this was in agreement with the kinetics release figures shown in Table 2. On the other hand, the mechanism of erosion was dominant in B3 formulation containing Eudragit^®^ (61.5%), especially at pH = 6.8. As previously mentioned, Eudragit^®^ L100-55 is a pH dependable multifunctional polymer, which is soluble at pH > 5.5 [62]. During the dissolution and erosion studies, it was observed that the B3 matrix tablets started to form a thick gel layer in acidic environment (pH = 1.2), which was followed by an abrupt matrix erosion in the neutral environment (pH = 6.8). Consequently, after the first 2 h of dissolution, a maximum release of the drug occurred, with a simultaneous total erosion of the tablets [63,64]. Formulations B4, B5 and B6 (containing 37.5%, 24% and 61.5% PEO, respectively) were characterized by a diffusion-based mechanism [65]. Ulvan and PEO combination matrix tablets (B7) were characterized also by a Fickian diffusion drug release. Both polymers created a cross-link network that entangled BUP, thus controlling its release [24].

Erosion studies on NTX tablet formulations N1 and N2 (which contained 58% and 37% HPMC, respectively) showed that HPMC augmented the swelling front of the tablets and delayed the water diffusion into the glassy core (optical observation) [60]. According to their kinetic profile (Korsmeyer–Peppas model), the mechanism of these matrix systems, was controlled by diffusion rather than erosion, *id est* that polymer chain relaxation was dominant comparatively to molecular diffusion [61]. Moreover, the hydration and erosion rate of N1 and N2 formulations was determined by the polymer combination used. In specific, in N1, which contained a higher amount of HPMC, it was initially observed a thicker gel layer than in N2, which, conversely, consisted of a higher amount of Eudragit^®^ leading to faster erosion [66]. Formulations N4 and N5 (58% and 37% PEO, respectively) showed a similarity in dissolution and consequently in the erosion profile. PEO when used in combination with Eudragit^®^ L100-55 produced a hydrogel layer, contributing mainly to a diffusional than erosive mechanism [67], which corresponded to a Fickian diffusion. Eudragit^®^ enhanced the erosion of the matrix, compared to N6 (95% PEO), contributing to a higher drug release. Drug release was characterized by a diffusion-based mechanism when PEO was used in a maximum quantity in formulation N6 (95%). For the first two hours, as the tablets remained in the acidic environment (pH = 1.2) a gel layer started forming, since PEO is a water-soluble excipient. After 4 h of dissolution and when the pH of the media was changed to neutral (pH = 6.8), the swelling and erosion front was in a synchronization, concomitantly conducting in a progressive erosion until the end of the dissolution experiment [65,68]. Matrix tablets N7 consisted of a PEO and ulvan combination; in this case the drug content was released in a Fickian pattern. Ulvan is characterized by a great capability of water solubility and jellification, physicochemical properties that are critical in the diffusional mechanism of the swelling matrix. In this matrix system combination, not only PEO, but also ulvan, created a cross-link network of polymers that entrapped the API and controlled its release in an extended way, leading after 8 h to complete erosion [24].

## 4. Discussion

Both bupropion and naltrexone have a pharmacological potential in the nervous system. Bupropion stimulates hypothalamic pro-opiomelanocortin (POMC) neurons’ activity, secreting *α*-melanocyte-stimulating hormone (*α*-MSH), which is related with lower energy intake and food craving. On the other hand, naltrexone acts in a synergistical way with bupropion by blocking *μ*-opioid receptors on POMC neurons resulting to a reduced release of *α*-MSH [69]. The efficacy of this combination treatment is examined by four clinical trials that are in phase three, named as CONTRAVE Obesity Research (COR), which use a modified intent-to-treat (ITT) analysis as well as attrition (COR-1,4 COR-II,5 COR-BMOD6 and COR-Diabetes) [70]. As shown in these four clinical trials naltrexone/bupropion combination has a great efficacy for the treatment of obesity, as it provides a better patient’s safety profile, in contrast with other medications (e.g., phentermine/topiramate), its use is well tolerated with common adverse effects and no abuse phenomena are provoked [69,71].

Moreover, the pharmacological and pharmacokinetic profiles of the two APIs were examined in order to ameliorate and to develop pharmaceutical forms simulating the commercially available [72,73]. As far as BUP·HCl is concerned, available cytotoxicity data have shown that less than 10 g are considered toxic [74] and the optimum dosage for extended release is 150 to 450 mg/day [72]. According to these data, the dosage used, was selected to be 75 mg (half of Wellbutrin^®^ XR 150 mg), as 75 mg is considered initiative to therapy [3]. In addition, NTX·HCl toxicity studies have shown that generally ≥1000 mg/kg can provoke mortalities and the optimum dosage is adjusted from 25 to 50 mg, depending on the stage of treatment [75]. However, in the present study, the therapeutic dosage was selected to be 8 mg, as it is considered effective in managing obesity, when used in combination with BUP·HCl [76].

**Figure 6:**
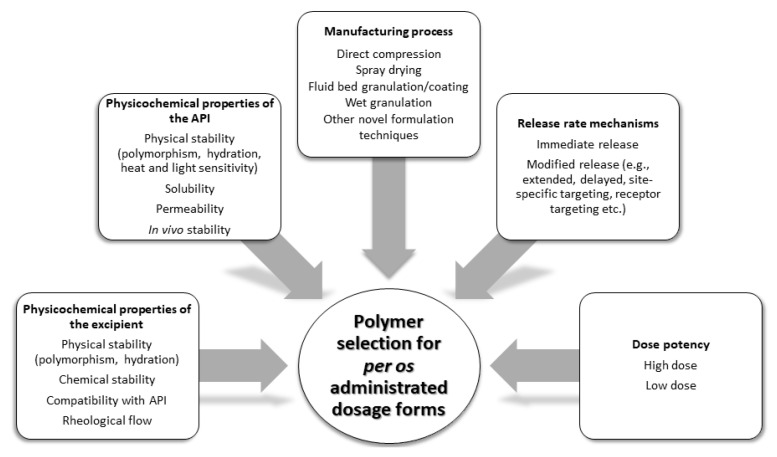
Parameters that are essential on selecting polymers for per o*s* administrative dosage forms.

The design of the aforementioned formulations was based on literature reports, which examine the properties of excipients in sustained release formulations. The properties of each excipient and the APIs, such as physicochemical properties (solubility, polymorphic structures, etc.), are also included for a more comprehensive and complete perception of the formulation design (Figure 6) [77]. Furthermore, it is well documented, and reported in the experimental analysis, that the utilized excipients are pharmacologically inactive and do not present any incompatible properties to those of the APIs, when formulated in matrix-type tablets [33,47].

**Figure 7:**
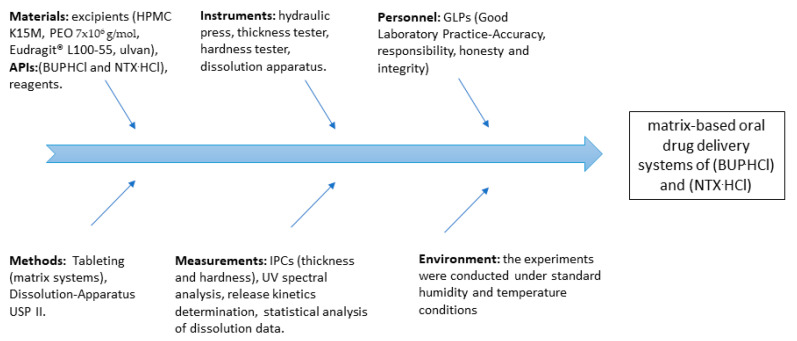
Ishikawa diagram: parameters that are crucial for the design and the development of matrix-based oral drug delivery systems of BUP·HCl and NTX·HCl.

The choice of excipients is based in several drug release and erosion studies that have shown a well-functionality and efficacious properties of the used polymers. These polymer-based drug delivery systems appeared to be depended by diffusion or erosion mechanisms, solvent activation (swelling or osmotically controlled) or stimulated by external factors (e.g., pH, temperature or sink conditions) [11,19,78] Moreover, an Ishikawa diagram or a “cause and effect diagram” (Figure 7), depicts concisely a lab scale manufacturing process, showing the most crucial factors and parameters, which contribute in the initial design and development of sustained release rate matrix-based oral drug delivery systems of BUP·HCl and NTX·HCl. Parameters such as materials, methods, instruments, measurements, personnel and environment are considered significant for the formulation process optimization [79].

Eudragit^®^ L100-55 is a soluble and pH dependable polymer, due to its ionized side group. It is characterized as a useful excipient in flavor masking and in coating (preventing moisture and light degradation of the API) [80]. Its use is very common when designing tablet formulations for targeted gastrointestinal delivery. In specific, Eudragit^®^ L100-55 is not soluble at acidic pH environments; however, it dissolves at pH > 5.5 in the small intestine [62]. Eudragit^®^ L100-55 enhances the release of BUP·HCl at pH 6.8. Additional studies indicated that the use of this polymer promotes an erosion mechanism of release at neutral pH. However, when Eudragit^®^ L100-55 is used in NTX·HCl formulations, a higher release of the active substance is observed in timepoints, which correspond to acidic conditions. This is assignable to the use of a large amount of Eudragit^®^ L100-55, in association with its water solubility properties, resulting in augmented water penetration in the matrix [58].

Another polymer, which is widely used in pharmaceutics, is PEO; it is characterized as a tablet binder, when used at concentrations of 5–85% and has excellent mucoadhesive properties. Moreover, polyethylene oxides of high molecular weight grades provide a delayed drug release via the hydrophilic matrix approach [81,82,83]. This polymer can form strong hydrogen bonds with water molecules thus leading to high water uptake and formation of a gel layer. In this report, its unique swelling/erosion properties were utilized in order to modify the release profiles of the two APIs. The use of PEO led to a decreased drug release, which could be due to the formation of a viscous gel layer. Erosion studies indicated a Fickian diffusion-based drug release mechanism.

One more water-soluble polymer is HPMC. It shows more extensive swelling properties and a steady rate of erosion of the polymeric matrix, after contact with gastrointestinal fluids, due to its slow water intake and small porosity characteristics. In particular, it is widely used in controlled release systems in order to provide a gradual, slow and extensive release rate of the active substance from the dosage form [84]. In this report, the HPMC K15M-based formulations were found effective in controlling the release of the highly water-soluble drug (BUP·HCl) and the sparingly water-soluble drug (NTX·HCl), due to rigid gel formation. When HPMC is used, as an excipient in these formulations, the mechanism of drug release is controlled by diffusion rather than erosion.

Ulvans are polysaccharides, which can be used in matrix monolithic systems, in order to provide a controlled release rate of the API, due to gel formation [85]. This property renders it a suitable excipient for exploiting its use in *per os* administered solid dosage forms. The results of our study revealed that when used in the produced tablets, this natural biopolymer created a hydrogel layer, which surrounded the formulations and allowed a slow and prolonged release of the APIs through the matrix.

## 5. Conclusions

Aiming at avoiding burst release of bupropion hydrochloride, and especially naltrexone hydrochloride, from oral tablets, and to concurrently achieve their controlled initial release, new formulations of these two drugs were prepared. The use of these modified release formulations, employing HPMC K15M, Eudragit^®^ L100-55, PEO (7 × 10^6^) and ulvan, as the rate controlling polymers, resulted in the relatively low release of these drugs, at 0 ≤ t ≤ 120 min. Under in vivo conditions this could possibly prove to aid the elimination of the peaks associated with daily drug administration, while maintaining continuous therapeutic levels for an extended time frame. This could decrease the possibility of appearance of adverse events, associated with peaks, and improve efficacy by avoiding drug concentration dunking. We are currently involved with the design and preparation of compounded bupropion hydrochloride and naltrexone hydrochloride tablets, *en route* to our future task to develop new oral formulations capable of confronting the obesity menace.

## Figures and Tables

**Table 1:** Composition of BUP·HCl and NTX·HCl tablet formulations (mg).

Ingredients	B1	B2	B3	B4	B5	B6	B7	N1	N2	N3	N4	N5	N6	N7
BUP HCl	75	75	75	75	75	75	75	-	-	-	-	-	-	-
NTX HCl	-	-	-	-	-	-	-	8	8	8	8	8	8	8
Eudragit^®^ L100-55	48	75	123	48	75	-	-	74	116	190	74	116	-	-
HPMC K15M	75	48	-	-	-	-	-	116	74	-	-	-	-	-
PEO (7 × 10^6^)	-	-	-	75	48	123	75	-	-	-	116	74	190	116
Ulvan	-	-	-	-	-	-	48	-	-	-	-	-	-	74
Mg. Stearate	2	2	2	2	2	2	2	2	2	2	2	2	2	2
*TOTAL*	200	200	200	200	200	200	200	200	200	200	200	200	200	200

**Table 2:** Kinetic release properties of the developed formulations (B1-B7, N1-N7), Wellbutrin^®^ XR 150 and 300 mg.

Formulations	MDT	t_20%_	t_50%_	t_90%_	*n*	Mean % D.E.
**B1**	98.86	37	284	*	0.36	39.56
**B2**	129.50	65	350	*	0.44	36.53
**B3**	194.58	138	210	399	**	51.97
**B4**	133.56	23	114	315	0.61	71.03
**B5**	145.93	32	137	393	0.61	62.81
**B6**	146.72	35	159	*	0.66	55.76
**B7**	144.99	45	167	*	0.67	56.75
**Wellbutrin^®^ XR 150 mg**	232.29	173	320	*	0.51	33.73
**Wellbutrin^®^ XR 300 mg**	217.44	163	354	*	0.75	31.74
**N1**	101.14	16	72	271	0.33	78.74
**N2**	78.93	13	70	269	**	83.67
**N3**	50.60	10	24	131	**	89.42
**N4**	124.16	16	98	342	0.31	72.21
**N5**	113.18	14	94	318	0.29	75.28
**N6**	169.97	46	283	*	0.56	45.60
**N7**	130.82	15	118	346	0.39	70.23

* The active pharmaceutical ingredient (API) did not reach 90% release during the dissolution experiment. ** Not Applicable.

## Supplementary Materials

The following are available online at https://www.mdpi.com/article/10.3390/polym13091456/s1, Table S1: title f_1_ and f_2_ indices of the developed formulations (B1-B7, N1-N7), Wellbutrin^®^ XR 150 and 300 mg; Table S2: ANOVA results of the developed formulations (B1-B7), Wellbutrin XR^®^ 150 and 300 mg; Table S3: ANOVA results of the developed formulations (N1-N7).
