# Supplementary material for: Probing the Release of Bupropion and Naltrexone Hydrochloride Salts from Biopolymeric Matrices of Diverse Chemical Structures
Source: Polymers (Basel). 2021 Apr 30;13(9):1456. doi: 10.3390/polym13091456 (PMC8125139; doi:10.3390/polym13091456)
Supplement: Supplementary file 1 [file polymers-13-01456-s001.zip › polymers-1182477-supplementary.pdf]

polymers

MDPI

Article

Modified release of bupropion and naltrexone hydrochloride salts from diverse matrix dosage forms

Angeliki Siamidi, Aikaterini Dedeloudi and Marilena Vlachou*

Division of Pharmaceutical Technology, Department of Pharmacy, School of Health Sciences, National and Kapodistrian University of Athens, 15784, Athens, Greece; asiamidi@pharm.uoa.gr (A.S.); dedeloud@pharm.uoa.gr (A.D.); vlachou@pharm.uoa.gr (M.V.)

* Correspondence: vlachou@pharm.uoa.gr; Tel.: +30-2107274674 (M.V.)

Supplementary material

Table S1. f_1 and f_2 indices of the developed formulations (B1-B7, N1-N7), Wellbutrin® XR 150 and 300 mg

FORMULATIONS	f_1	f_2	FORMULATIONS	f_1	f_2
B1 vs B2	10.02	65.24	N1 vs N2	10.84	54.61
B1 vs B3	51.32	28.97	N1 vs N3	21.45	36.30
B1 vs B4	38.33	25.63	N1 vs N4	12.57	53.78
B1 vs B5	34.21	27.25	N1 vs N5	6.42	66.39
B1 vs B6	26.51	34.47	N1 vs N6	92.45	22.96
B1 vs B7	24.37	36.28	N1 vs N7	20.19	45.41
B1 vs Wellbutrin® XR 150mg	58.93	32.20	N2 vs N3	11.90	47.75
B1 vs Wellbutrin® XR 300mg	52.54	34.18	N2 vs N4	27.75	40.47
B2 vs B3	48.04	29.51	N2 vs N5	19.84	46.01
B2 vs B4	44.72	23.60	N2 vs N6	124.43	20.29
B2 vs B5	40.04	25.45	N2 vs N7	38.13	35.43
B2 vs B6	33.02	32.07	N3 vs N4	45.01	29.59
B2 vs B7	29.53	34.25	N3 vs N5	36.03	32.56
B2 vs Wellbutrin® XR 150mg	48.29	36.45	N3 vs N6	154.75	15.60
B2 vs Wellbutrin® XR 300mg	39.82	39.29	N3 vs N7	56.79	26.42
B3 vs B4	31.96	30.45	N4 vs N5	4.74	69.51
B3 vs B5	26.25	33.78	N4 vs N6	65.40	27.15
B3 vs B6	30.44	36.02	N4 vs N7	6.28	64.45
B3 vs B7	28.01	39.16	N5 vs N6	73.63	24.62
B3 vs Wellbutrin® XR 150mg	66.88	32.98	N5 vs N7	11.57	53.46
B3 vs Wellbutrin® XR 300mg	76.40	30.66	N6 vs N7	33.53	29.54
B4 vs B5	9.35	59.13			
B4 vs B6	22.21	43.00			
B4 vs B7	29.01	39.72			
B4 vs Wellbutrin® XR 150mg	145.28	19.40			
B4 vs Wellbutrin® XR 300mg	159.27	18.62			
B5 vs B6	11.71	53.92			
B5 vs B7	17.54	48.18			
B5 vs Wellbutrin® XR 150mg	100.75	22.19			
B5 vs Wellbutrin® XR 300mg	115.28	21.02			
B6 vs B7	5.22	72.08			
B6 vs Wellbutrin® XR 150mg	79.71	26.57			
B6 vs Wellbutrin® XR 300mg	92.71	25.62			
B7 vs Wellbutrin® XR 150mg	70.80	29.12			
B7 vs Wellbutrin® XR 300mg	83.16	27.98			
Wellbutrin® XR 150mg vs Wellbutrin® XR 300mg	13.17	60.63			

Table S2. ANOVA results of the developed formulations (B1-B7), Wellbutrin XR® 150 and 300 mg

One-way analysis of variance			
P value	< 0.0001		
P value summary	***		
Are means signif. different? (P < 0.05)	Yes		
Number of groups	9		
F	0,7780		
R squared	0,0007884		
ANOVA Table	SS	df	MS
Treatment (between columns)	9349	8	1169
Residual (within columns)	76.47	40	1.912
Total	9433	26	

Tukey's multiple comparisons test	Mean Diff,	95,00% CI of diff,	Significant?	Summary
B1 vs. B2	3,170	1,399 to 4,941	Yes	**
B1 vs. B3	-12,07	-16,45 to -7,690	Yes	***
B1 vs. B4	-31,14	-35,94 to -26,35	Yes	****
B1 vs. B5	-22,92	-30,25 to -15,59	Yes	***
B1 vs. B6	-15,97	-24,54 to -7,402	Yes	**
B1 vs. B7	-16,62	-20,54 to -12,70	Yes	****
B1 vs. WB150	6,238	0,9194 to 11,56	Yes	*
B1 vs. WB300	8,228	2,909 to 13,55	Yes	**
B2 vs. B3	-15,24	-17,85 to -12,63	Yes	****
B2 vs. B4	-34,31	-37,34 to -31,29	Yes	****
B2 vs. B5	-26,09	-31,74 to -20,43	Yes	****
B2 vs. B6	-19,14	-25,97 to -12,32	Yes	***
B2 vs. B7	-19,79	-22,06 to -17,52	Yes	****
B2 vs. WB150	3,068	-0,4800 to 6,617	No	ns
B2 vs. WB300	5,058	1,510 to 8,607	Yes	*
B3 vs. B4	-19,07	-19,51 to -18,63	Yes	****
B3 vs. B5	-10,85	-14,22 to -7,476	Yes	***
B3 vs. B6	-3,900	-8,195 to 0,3946	No	ns
B3 vs. B7	-4,548	-5,820 to -3,276	Yes	***
B3 vs. WB150	18,31	17,37 to 19,25	Yes	****
B3 vs. WB300	20,30	19,36 to 21,24	Yes	****
B4 vs. B5	8,223	5,078 to 11,37	Yes	***
B4 vs. B6	15,17	11,31 to 19,03	Yes	****
B4 vs. B7	14,52	12,99 to 16,05	Yes	****
B4 vs. WB150	37,38	36,83 to 37,93	Yes	****
B4 vs. WB300	39,37	38,82 to 39,92	Yes	****
B5 vs. B6	6,947	3,621 to 10,27	Yes	**
B5 vs. B7	6,298	2,154 to 10,44	Yes	**
B5 vs. WB150	29,16	26,45 to 31,86	Yes	****
B5 vs. WB300	31,15	28,44 to 33,85	Yes	****
B6 vs. B7	-0,6483	-5,817 to 4,521	No	ns
B6 vs. WB150	22,21	18,80 to 25,62	Yes	****
B6 vs. WB300	24,20	20,79 to 27,61	Yes	****
B7 vs. WB150	22,86	20,94 to 24,77	Yes	****
B7 vs. WB300	24,85	22,93 to 26,76	Yes	****
WB150 vs. WB300	1,990		Yes	****

Table S3. ANOVA results of the developed formulations (N1-N7)

**One-way analysis of variance**			
P value	< 0.0001		
P value summary	***		
Are means signif. different? (P < 0.05)	Yes		
Number of groups	7		
F	0,4121		
R squared	0,0001225		
ANOVA Table	SS	df	MS
Treatment (between columns)	7089	6	1181
Residual (within columns)	12.66	30	0.4221
Total	7103	41	

Tukey's multiple comparisons test	Mean Diff,	95,00% CI of diff,	Significant?	Summary
N1 vs. N2	-4,898	-6,830 to -2,967	Yes	***
N1 vs. N3	-10,80	-11,74 to -9,860	Yes	****
N1 vs. N4	6,520	5,503 to 7,537	Yes	****
N1 vs. N5	3,453	1,820 to 5,086	Yes	**
N1 vs. N6	33,10	31,58 to 34,61	Yes	****
N1 vs. N7	8,870	5,504 to 12,24	Yes	***
N2 vs. N3	-5,902	-6,990 to -4,814	Yes	****
N2 vs. N4	11,42	9,818 to 13,02	Yes	****
N2 vs. N5	8,352	7,856 to 8,847	Yes	****
N2 vs. N6	38,00	37,42 to 38,57	Yes	****
N2 vs. N7	13,77	12,03 to 15,51	Yes	****
N3 vs. N4	17,32	16,28 to 18,36	Yes	****
N3 vs. N5	14,25	13,49 to 15,02	Yes	****
N3 vs. N6	43,90	43,15 to 44,65	Yes	****
N3 vs. N7	19,67	16,96 to 22,38	Yes	****
N4 vs. N5	-3,067	-4,522 to -1,612	Yes	**
N4 vs. N6	26,58	25,49 to 27,66	Yes	****
N4 vs. N7	2,350	-0,4230 to 5,123	No	ns
N5 vs. N6	29,64	29,01 to 30,28	Yes	****
N5 vs. N7	5,417	3,207 to 7,626	Yes	***
N6 vs. N7	-24,23	-26,24 to -22,21	Yes	****
